# Puzzling Out Synaptic Vesicle 2 Family Members Functions

**DOI:** 10.3389/fnmol.2017.00148

**Published:** 2017-05-22

**Authors:** Odile Bartholome, Priscilla Van den Ackerveken, Judit Sánchez Gil, Orianne de la Brassinne Bonardeaux, Pierre Leprince, Rachelle Franzen, Bernard Rogister

**Affiliations:** ^1^Laboratory of Nervous System Disorders and Therapy, GIGA-Neurosciences, University of LiègeLiège, Belgium; ^2^Department of Neurology, Centre Hospitalier Universitaire de Liège (CHU), University of LiègeLiège, Belgium

**Keywords:** SV2 protein, SV2 functions, neurotransmission, epilepsy, neurological diseases

## Abstract

Synaptic vesicle proteins 2 (SV2) were discovered in the early 80s, but the clear demonstration that SV2A is the target of efficacious anti-epileptic drugs from the racetam family stimulated efforts to improve understanding of its role in the brain. Many functions have been suggested for SV2 proteins including ions or neurotransmitters transport or priming of SVs. Moreover, several recent studies highlighted the link between SV2 and different neuronal disorders such as epilepsy, Schizophrenia (SCZ), Alzheimer’s or Parkinson’s disease. In this review article, we will summarize our present knowledge on SV2A function(s) and its potential role(s) in the pathophysiology of various brain disorders.

## Sv2: Basic Facts

SV2A belongs to the Synaptic vesicle (SV) protein 2 family, which also includes SV2B and SV2C. These three paralogs can be found in vesicles of neuronal or endocrine cells. All members are composed of a highly N-glycosylated 80 kDa backbone (Table 1) organized in 12 transmembrane (TM) regions. They also present two large loops—one cytoplasmic and one luminal—and N- and C-terminal cytoplasmic sequences (Janz et al., 1999). SV2A is the only member of the SV2 family ubiquitously expressed in the adult brain, and is also found in neuroendocrine cells and at neuromuscular junctions (Buckley and Kelly, 1985; Bajjalieh et al., 1994; Dong et al., 2006).

**Table 1:** **SV2 protein family information summary**.

	SV2A	SV2B	SV2C
Gene	Chromosome 3	Chromosome 7	Chromosome 13
	14,371 bp	194,369 bp	177,984 bp
mRNA	4334 bp	5307 bp	9101 bp
	13 exons	13 exons	13 exons
Protein	82 kD	77 kD	82 kD
	742 a.a.	683 a.a.	727 a.a.
Expression	Whole brain	Whole brain except	Central neuraxis
		DG, GP, RNT, SN	
Synapses types	GA, Gl	Gl	GA, D, C

*Genes, mRNAs and proteins information of murine SV2A, SV2B and SV2C (http://www.informatics.jax.org; http://vega.sanger.ac.uk). DG, *dentate gyrus*; GP, *globus pallidus*; RNT, the reticular nucleus of the thalamus; SN, *subtantia nigra*; GA, GABAergic; Gl, Glutamatergic; D, Dopaminergic; C, Cholinergic*.

Proteins of the SV2 family share approximately 60% of their sequences, especially around TM and C-terminal regions. Though luminal loops differ among SV2 proteins, they always contain at least three N-glycosylation sites and present a repeated structure of hydrophobic amino-acids (Janz and Sudhof, 1999). The SV2 family is well conserved among vertebrates but no invertebrate isoform has been found. However, SV2 proteins exhibit a topology related to other transporter proteins, as their first six TM domains are homologous to the major facilitator superfamily (MFS) to which the human glucose transporter belongs, and the other six are closer to neurotransmitter transporters (Feany et al., 1992).

Another homologous protein is SVOP (SVtwO-related Protein; Bajjalieh et al., 1992; Janz et al., 1998). SVOP is a non-glycosylated protein also found in SVs, rather distantly related to SV2 proteins (only 20%–22% homology) but with a similar structure. It also contains 12 TM domains, both termini regions are cytoplasmic, but SVOP does not feature either a long intraluminal loop or glycosylated sites. Unlike SV2 proteins, SVOP is evolutionarily conserved in vertebrates and invertebrates. The role of SVOP is currently unknown but its absence in *SVOP* KO mice does not lead to an abnormal phenotype (Yao et al., 2013).

Even if SV2 proteins share the majority of their sequences, they all have specific expression patterns. As previously said, SV2A is found in all areas of the brain, including the trigeminal nuclei (Edvinsson et al., 2015) and the sphenopalatine ganglion (Steinberg et al., 2016) where it was recently found. SV2B follows the same pattern of expression with the exception of *dentate gyrus (DG)*, *globus pallidus (GP)*, the reticular nucleus of the thalamus and the reticular part of the *substantia nigra* (Bajjalieh et al., 1994; Crèvecœur et al., 2013). Finally, SV2C has a more variable expression. It is present in the central neuraxis, including the striatum, midbrain (especially in the *substantia nigra*), and hindbrain at high level. Low levels of SV2C can be found in the cerebrum, the olfactory bulb, the hippocampus and the cerebellum (Dardou et al., 2011). A closer look at certain structures of the brain emphasizes another difference between all paralogs: SV2A can be found in glutamatergic and GABAergic neurons, in all cortical layers of the cerebellum for example. On the other hand, SV2B seems to be restricted to some glutamatergic neurons, as it is found in granule cells of the cerebellum, but is absent from *DG* granule cells (Bajjalieh et al., 1994; Crèvecœur et al., 2013). SV2C is found in certain GABAergic cell types, like Purkinje cells of the cerebellum, in dopaminergic neurons as well as a fraction of cholinergic neurons (Dardou et al., 2011). The specificity of SV2B and SV2C for respectively glutamatergic and GABAergic synapses was also observed by proteomic analysis and co-localization study of immuno-isolated vesicles (Grønborg et al., 2010; Bragina et al., 2012).

During the mouse development, the expression pattern of SV2A and SV2B starts at mid-neurogenesis, at embryonic day 14 (E14). Distribution of SV2A stabilizes around birth (postnatal day 0—P0) while that of SV2B seems to be more variable during life (Bajjalieh et al., 1994; Crèvecœur et al., 2013). Between P5 and P7, the amount of SV2A greatly increases specifically in the CA1 region of the hippocampus, that of SV2B gradually raises from P5 to P10 in the same region while that of SV2C does not change (Crèvecœur et al., 2013). The expression of SV2C has not been the subject of a detailed study.

The correct folding of SV2A appears to be important for its function and proper location since many substitution mutations give rise to diffuse or aggregated cytoplasmic locations of SV2A (Chang and Sudhof, 2009; Nowack et al., 2010; Yao et al., 2010; Kwon and Chapman, 2012). The exact role of N-glycosylation of SV2 proteins is unknown. However, two SV2A mutants (N498Q/N573Q or N548Q/N573Q double mutations) displaying diffuse cytoplasmic localization, those N-glycosylation sites could be implicated in folding or quality control in the endoplasmic reticulum (Kwon and Chapman, 2012). For SV2B and SV2C, glycosylation seems to be even more crucial as mutants with only one modified glycosylation site (SV2B-N516A and SV2C-N559A) show a reduction of expression whereas SV2A-N573A shows no phenotype (Yao et al., 2016).

Little is known about the regulation of *SV2A* gene expression. Prediction analysis by SABiosciences’ Text Mining Application and UCSC’s Genome Browser identified several potential transcription factor binding sites present on *SV2A* promoter, but the functional importance of any of them has still to be proven. Likewise, more than 70 binding sites for microRNAs are potentially located on the *SV2A* mRNA sequence according to StarBase®, but only one of them has been studied in detail. MiR-485 is present in the hippocampus at least between E18.5 and P20 and is able *in vitro* to specifically decrease SV2A levels without affecting other synaptic proteins. Overexpression of miR-485 or SV2A siRNA-mediated knockdown produce similar results: a reduction of dendritic spine density, clustering of PSD-95 and surface expression of GluR2 (Cohen et al., 2011). However, other studies did not show any change in synaptic density in *SV2A* KO mice nor in *SV2A* KO cortical neurons (Crowder et al., 1999; Custer et al., 2006).

## Sv2A: Functions

### Effect on Neurotransmission

One of the first trials on the role of SV2A was the extreme phenotype displayed by *SV2A* KO mice. Mice that survive birth seem to be normal during their first days of life. However, after 1–2 weeks, they experience seizures, quickly followed by weight loss and death around P20 (Crowder et al., 1999; Janz et al., 1999). Importantly, seizures in this KO model seem to be more extreme than those observed in other synaptic protein mutant mice (Janz et al., 1999). Since the whole brain architecture is not modified, with no changes in synapse morphology or density, it appears that the brain develops correctly, making a developmental role for SV2A unlikely (Crowder et al., 1999; Janz et al., 1999). On the contrary, *SV2B* KO mice are viable with no apparent phenotype and *SV2A*/*SV2B* double KO (DKO) mice display the same phenotype as *SV2A* KO (Janz et al., 1999).

**Figure 1:**
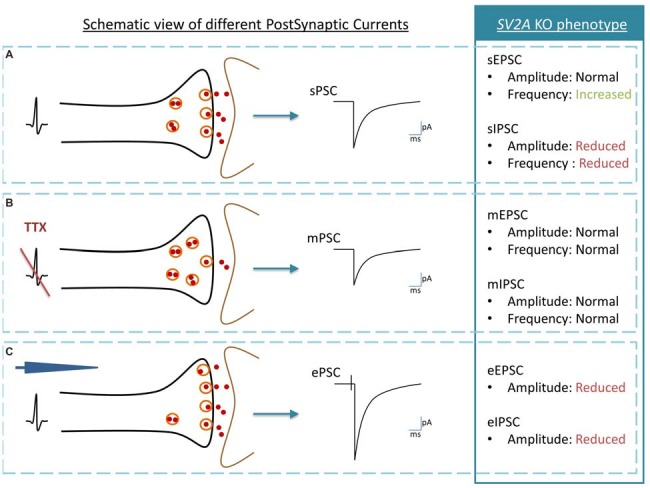
**Schematic view of post synaptic currents (PSC) and summary of Synaptic vesicle proteins 2A (SV2A) KO phenotype. (A)** Spontaneous PSC or sPSC: an action potential in the presynaptic neuron (black) induces a sPSC in the postsynaptic neuron (brown). **(B)** Miniature PSC or mPSC: the generation of action potentials is blocked by Tetrodotoxin (TTX). If SVs exocytosis is functional, a single vesicle is release by the presynaptic neuron (black) and induces a mPSC in the postsynaptic neuron (brown). The mPSC reveals thus the “molecular machinery” of SV exocytosis while sPSC also reveals the link between vesicle exocytosis and the pre-synaptic action potential. **(C)** Evoked PSC or evoked postsynaptic currents (ePSC): an action potential is induced with an electrode (blue) in the presynaptic neuron (black). The postsynaptic neuron (gray) responds by producing an ePSC. In comparison to the sPSC, the ePSC recruits the maximal possibilities of SVs exocytosis. The analysis of SV2A KO phenotype is telling us that: (1) in physiological conditions, inhibitory PSC are affected negatively by the SV2A absence, both in amplitude and in frequency although excitatory PSC are modified only through increased frequency; (2) the absence of SV2A does not interfere with the molecular mechanism of the SV exocytosis process; and (3) when the synapse is pushed at its maximum of activity, both excitatory and inhibitory PSC are decreased in amplitude.

From an electrophysiological point of view, *SV2A* KO mice hippocampi display normal miniature Excitatory and Inhibitory Postsynaptic Currents (mEPSC and mIPSC) frequencies. As mPSC are inputs observed without action potentials thus accounting for the response obtained after the release of a single vesicle, this excludes a role of SV2A in vesicle fusion events (Crowder et al., 1999; Janz et al., 1999; Chang and Sudhof, 2009; Venkatesan et al., 2012). In contrast, spontaneous postsynaptic currents (sPSC, the multiple vesicles event obtained without any experimental stimulation of a cell) and evoked postsynaptic currents (ePSC, the multiple vesicles event response of a cell obtained after experimental stimulations) are affected in *SV2A* KO (Figure 1). While all studies agree that the absence of SV2A induces a reduction of the frequency and amplitude of inhibitory postsynaptic currents (IPSC), either spontaneous or evoked (Crowder et al., 1999; Chang and Sudhof, 2009; Venkatesan et al., 2012), its effect on excitatory postsynaptic currents (EPSC) is still discussed. Recording made on primary cultured hippocampal autapses from *SV2A*/*SV2B* DKO mice shows smaller amplitudes of eEPSC compared to *SV2B* KO autapses. Following observations of a facilitation response of *SV2A*/*SV2B* DKO neurons during trains of action potentials and a smaller readily releasable pool of vesicles (RRP), the author hypothesized that the function of SV2A is linked to the release probability (Custer et al., 2006). These results are in agreement with a previous study performed on chromaffin cells showing a reduction of calcium-induced exocytotic bursts and of SDS resistant SNARE complexes in absence of SV2A, resulting in a smaller RRP (Xu and Bajjalieh, 2001). Those two studies argue for an involvement of SV2A in vesicle fusion competence (Xu and Bajjalieh, 2001; Custer et al., 2006). Still, another study shows that cultured primary cortical neurons from *SV2A*/*SV2B* DKO display synaptic facilitation during stimulus trains but no RRP changes (Chang and Sudhof, 2009). These authors thus exclude a priming role for SV2A but rather argue for a Ca^2+^-responsiveness enhancer role in primed vesicles (Chang and Sudhof, 2009). A more recent electrophysiological study performed on hippocampal slices showed that in the absence of SV2A, EPSC amplitudes remain unchanged while the associated frequencies increase (Venkatesan et al., 2012). The author emphasized that the opposite effect of SV2A on excitatory and inhibitory currents observed in hippocampal slices of *SV2A* KO likely underlines an effect of SV2A on GABAergic network subsequently impacting the Glutamatergic synapses rather than a direct effect of SV2A on both networks. Interestingly, overexpression of SV2A in autaptic hippocampal neurons produced the typical neurotransmission defect observed in *SV2A* KO mice (Nowack et al., 2011).

**Figure 2:**
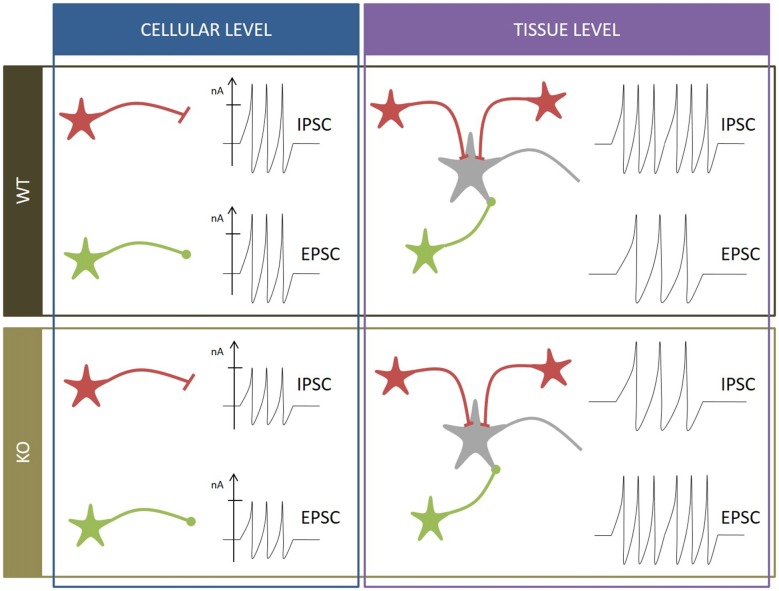
**Summary of neurophysiological observations**. WT condition (black box): at cellular level (blue box), inhibitory postsynaptic currents (IPSC) and EPSC of inhibitory neurons (in red) and excitatory neurons (in green) display characteristic amplitudes and frequencies. At tissue level (purple box), the inhibitory frequency is higher than the excitatory frequency to moderate global input. *SV2A* KO condition (brown box): at cellular level (blue box), amplitudes of IPSC and EPSC are reduced. At tissue level (purple box), the excitatory frequency is higher than the inhibitory frequency, leading to seizure onset and epilepsy.

Although at first sight all these results do not seem to fit together correctly, a closer look at all of them could help raise a hypothesis about the role of SV2A (Figure 2). Custer, Chang and Sudhof measured electrophysiological parameters in neurons cultivated from WT and KO animals. Proportionally, differences of IPSC and EPSC amplitudes between WT and KO are similar (±37% for EPSC, ±39% for IPSC), which could suggest that they result from the same defect. But each system has its own characteristics. Inhibitory synapses are usually placed closer to the soma than excitatory synapses (Ito et al., 1997; Gulyás et al., 1999; Magee and Cook, 2000; Megías et al., 2001; Darstein et al., 2003; Kawakami et al., 2003; Kulik et al., 2003; Gassmann et al., 2004), which implies that inhibitory signals are thus more constant and control the traffic of excitatory local responses (Jaffe and Carnevale, 1999). Moreover, at least in the hippocampus, inhibitory synapses tend to be more robust in contrast to excitatory synapses, which compensate failure occurrences by a burst firing pattern (Miles and Wong, 1984, 1986). Those observations suggest that the inhibitory system could be more sensitive to SV2A depletion and potentially explain Venkatesan’s results: at cellular level, the amplitude of both PSC is reduced due to the absence of SV2A but at tissue level, the impact on the inhibitory system is overriding. Lack of inhibitory currents cancels its restraining role and unleashes excitatory currents, leading to an overload of the excitatory defect and a conversion to overall hyperactivity. This hypothesis doesn’t rule out that SV2A might somehow be linked to Ca^2+^-dependent transmissions in both glutamate and GABA networks.

### Transporter

Due to the evident structural links between SV2A and different transporters, it was first proposed that SV2A was either a neurotransmitter-, an ion- or a glucose-transporter. Interestingly, 3D reconstructions and structural studies based on MFS models show that SV2A has two different conformations with a central depression facing towards one or the other side of the membrane, strengthening the possibilities of a transporter activity (Lynch et al., 2008; Lee et al., 2015). The neurotransmitter transporter hypothesis is rather unlikely because SV2A is found in GABA and glutamate releasing synapses (Bajjalieh et al., 1994) and is present in immune-isolated VGlut1-, VGlut2- and VGat-containing vesicles, although its concentration may be higher in VGat-containing vesicles (Grønborg et al., 2010; Bragina et al., 2012). Moreover, quantal and vesicle sizes are normal in *SV2A* KO, suggesting that neurotransmitter transport is unaffected (Xu and Bajjalieh, 2001; Custer et al., 2006). Concerning ion transport, the early identification of two negatively charged residues in the first TM region raised the possibility that SV2A could transport Ca^2+^. This issue was also ruled out when neither the overexpression nor the silencing of *SV2A* induced a modulation of cytosolic or intravesicular Ca^2+^ concentration in INS-1E insulinoma cells (Iezzi et al., 2005), although those measurements were obtained in a non-physiological system. Furthermore, these results are in agreement with previous data showing that the calcium-dependent release is not modified in *SV2A* KO chromaffin cells (Xu and Bajjalieh, 2001). On the contrary, SV2B could perform such function as its absence in cultured rod cells of the retina induces an increase in presynaptic Ca^2+^ concentration (Wan et al., 2010). Finally, the fact that insulin release is decreased after *SV2A* or *SV2C* silencing in INS-1E cells (Iezzi et al., 2005) is an argument in favor of a glucose receptor function. A recent study shows that the expression of human SV2A in hexose transport-deficient yeast restored the growing capacity on a galactose-only medium. Moreover, a significant uptake of galactose could be quantified in human SV2A-expressing yeasts (Madeo et al., 2014). The role of carbon hydrates has been increasingly noted over the last decade. Indeed, carbohydrates, including glucose and galactose, contribute to the development but also to proper functions of the nervous system through glycosaminoglycans, which are implicated in proteoglycan (PG) formation. PG could have a role in axon guidance, pathfinding, and synapse formation but also in plasticity, or injury recovery (Zhang et al., 2006; Um and Ko, 2013; Silver and Silver, 2014; Smith et al., 2015; Takeda-Uchimura et al., 2015). In this regard, SV2A as hexose transporter could play a crucial role in the nervous system.

### Gel Matrix

Although SV2A may not be a neurotransmitter transporter, it could play a role in neurotransmitter concentration and release by forming an intravesicular gel matrix. Indeed, in mast cells, it has been shown that serotonin is restrained in vesicles by an intragranular matrix, slowing its mobility and limiting its release more than pore diameters would (Alvarez de Toledo et al., 1993; Nanavati and Fernandez, 1993; Marszalek et al., 1997). Actually, acetylcholine and ATP found in SVs of the *Torpedo* electric organ seem to be fully released only when challenged with high NaCl or KCl concentrations, suggesting the existence of a charged matrix (Reigada et al., 2003). SV2A being the main Keratan-Sulfate proteoglycan (KS-PG) of those SVs with all glycans located in their lumen (Feany et al., 1992; Scranton et al., 1993), could be a likely candidate to support this role. Moreover, SV2A needs at least one N-glycosylation site available to be correctly targeted to synapses (Kwon and Chapman, 2012), indicating that glycans appear to be necessary for a proper SV2A function even if their exact role in this matter needs to be defined. In addition, isolated SVs filled with glutamate are bigger than empty ones, and those lacking SV2A do not present such change (Budzinski et al., 2009). However, previous studies have shown that SVs from *SV2A* KO mice do not differ in size compared to those in WT (Crowder et al., 1999; Janz et al., 1999; Custer et al., 2006). The fact that Budzinski et al. (2009) work on isolated SVs could explain this difference in their observations. In the absence of further inputs on this matter, it is difficult to conclude anything regarding this hypothesis.

### SV2A and Synaptotagmin

Bennett et al. (1992) immunoprecipitated SV membrane proteins by different methods and shed light on possible interactions that SV2A might have with other membranous synaptic proteins. Using three different detergents (CHAPS, octylglucoside and Triton X-100), they showed that SV2A could be in a complex with synaptotagmin 1 (Syt1 or p65), synaptophysin (Syp or p38) or Ras-related protein (rab3A; Bennett et al., 1992). Since then, a lot of efforts have been made to identify protein species that bind to SV2A. While little evidence has been found in favor of a real interaction between Syp or rab3A and SV2A, the link between Syt1 and SV2A has rapidly been established. Despite early studies showing an absence of interaction between SV2A and Syt1 (Chapman et al., 1998; Littleton et al., 1999), it is now clear that all SV2 paralogs bind to Syt1 (Schivell et al., 1996, 2005; Lazzell et al., 2004).

In the pre-synaptic region, synaptotagmin is found on SV and triggers the calcium-dependent exocytosis by binding Ca^2+^ on two C2 domains, quickly followed by the penetration of those domains into the targeted cellular membrane (Brose et al., 1992; Bai et al., 2002). This interaction of Syt1 with the plasma membrane induces a bending of the membrane, allowing the SV to complete fusion (Martens et al., 2007; Hui et al., 2009). Syt1 is found in most brain areas but is particularly expressed in rostral parts (Geppert et al., 1991; Xu et al., 2007).

It is interesting to note that the interaction of SV2 proteins with Syt1 seems highly controlled. First, the domain in all SV2 that binds Syt1 is negatively regulated by calcium as an increase in calcium concentration reduces the amount of SV2 immunoprecipitated with GST-Syt (Schivell et al., 2005). This observation corroborates the fact that the normalization of the elevated calcium concentration in *SV2B* KO rod cells restores normal neurotransmission (Wan et al., 2010). On the other hand, another study reports that the binding of SV2B to Syt1 in terminals of photoreceptor cells is not calcium-dependent. However, the range of tested concentrations was smaller compared to Wan’s studies, therefore the inhibitory effect could have been missed (Lazzell et al., 2004). The exact localization of the binding site is not known but should include residues 56–74, as antibodies directed against this region prevent the interaction between SV2B and Syt1 (Schivell et al., 1996, 2005). Second, SV2A and SV2C have an additional binding site that is positively regulated by calcium. This supplementary site could further modulate the interaction with Syt1 (Schivell et al., 2005). Third, the amino-termini of SV2A could be highly phosphorylated by members of the casein kinase 1 family, increasing the affinity of SV2A for Syt1 (Pyle et al., 2000). The N-terminal of SV2A features two phosphorylated clusters but only Threonine 84 of SV2A seems essential for binding since it forms hydrogen bonds with three conserved Lysine residues of the C2B domain of Syt1 (Zhang et al., 2015). Protein BLAST of SV2 proteins shows that this Threonine is conserved in all SV2 paralogs^1^.

While little evidence has been provided to confirm a role for SV2A in vesicle priming as discussed above, another hypothesis has emerged arguing that SV2A seems to have two functions regarding Syt1. Reduction or overexpression of SV2A induces a reduced or increased abundance of Syt1 respectively, implying that SV2A could regulate Syt1 expression and/or degradation. In addition, SV2A participates in the internalization of Syt1 as its absence raises Syt1 concentration in the cellular membrane (Yao et al., 2010; Nowack et al., 2011; Kaempf et al., 2015).

**Figure 3:**
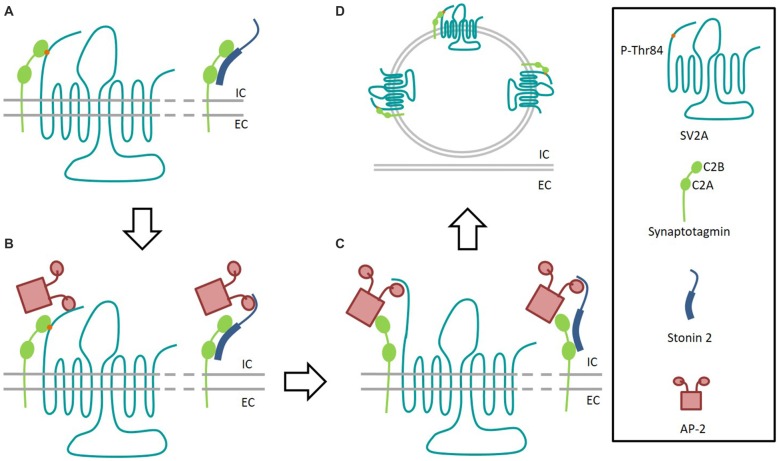
**Interaction model of SV2A and Stonin 2 with Synaptotagmin 1. (A)** After fusion event, the C2B domain of Synaptotagmin 1 (green) interacts with SV2A (light blue) phosphorylated-T84 residue (orange) and Stonin 2 (dark blue) binds to both C2 domains of Synaptotagmin to initiate recycling. **(B)** SV2A and Stonin 2 bind to AP-2 (pink) to facilitate the formation of the endocytosis complex. **(C)** Synaptotagmin 1 binds to AP-2. Dephosphorylated SV2A keeps interaction with AP-2 to consolidate the endocytosis complex. **(D)** After endocytosis, the fusion competent vesicle presents Synaptotagmin 1 bond again to T84-phosphorylated SV2A. IC, Intracellular; EC, Extracellular.

Interestingly, the mutation of one residue in the first endocytosis motifs in the amino-termini of SV2A does not affect the overall capacity of SV2A to bind Syt1, supporting the idea that the additional amino-binding site is not the main one (Yao et al., 2010). On the other hand, the same mutation shows a decrease of interactions with the clathrin adaptor AP-2 compared to wild-type. AP-2 is known to bind to Syt1 at residues close to those involved in SV2A binding (Haucke et al., 2000), and immunoprecipitation studies show that AP-2, Syt1 and SV2A can form a tripartite complex in rat brains (Haucke and De Camilli, 1999). Since the SV2A key-residue for Syt1 binding is Threonine 84 and the one potentially implicated in AP-2 binding is Tyrosine 46, SV2A could therefore be considered as an intrinsic trafficking partner (iTRAP) to facilitate AP-2 and Syt1 interactions (Figure 3; Gordon and Cousin, 2016). These observations underline the similarity between SV2A and another protein implicated in Syt1 retrieval, Stonin-2 (Stn2). Indeed, this protein is able to interact both with Syt1 (primarily by its C2A domain) and the ear domain of the α subunit of AP-2 (Diril et al., 2006). In such a model, either AP-2 would then bind Syt1 via its C2B domain forming a multimeric complex or Stn2 would deliver Syt1 to AP-2 (Jung et al., 2007).

Interestingly, while the mutation of *Stn2* ortholog in invertebrates (in which *SV2A* do not exist) is lethal (Fergestad et al., 1999; Mullen et al., 2012), it is not the case for *Stn2* KO mice which are perfectly viable and fertile (Kononenko et al., 2013). This fact is in agreement with the hypothesis that SV2A could be implicated in an alternative pathway to Syt1 recycling, required to safeguard or to refine the sorting of SV in the central nervous system of vertebrates (Kaempf et al., 2015). This possibility is strengthened by the fact that *SV2A* KO mice display elevated levels of Stn2, and that the combined absence of SV2A and Stn2 is responsible for an aggravated phenotype (Kaempf et al., 2015).

Nonetheless, the fact that certain SV2A mutants failed to rescue synaptic depression of neurotransmission observed in *SV2A* KO neurons, while displaying normal Syt1 concentration on the plasma membrane (Nowack et al., 2010), suggests that the Syt1 recycling role might not be primary for SV2A and that this protein could potentially perform other function(s) at the synapse.

### Other Leads on SV2A Functions

**Figure 4:**
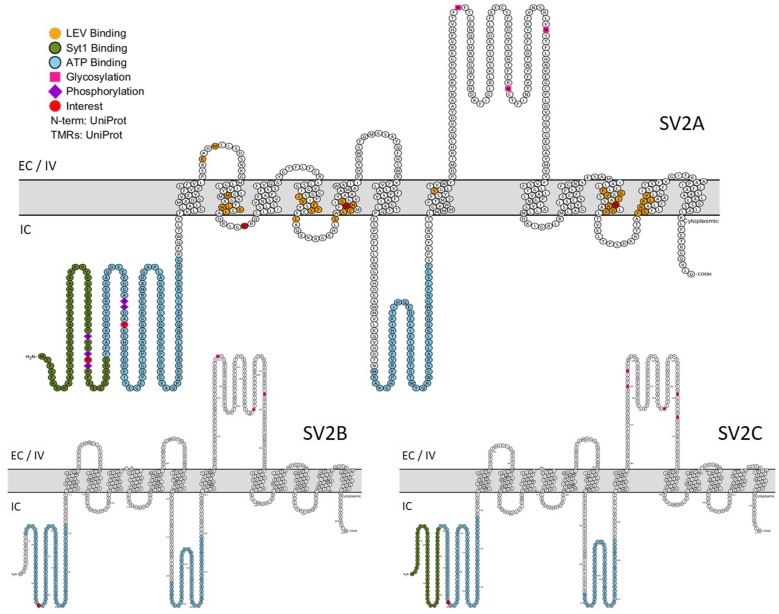
**Primary structures of SV2 proteins**. In green: Synaptotagmin 1 binding domain. In blue: nucleotide binding domains. In orange: residues implicated in LEV binding. Purple lozenge: phosphorylated residues. Pink square: glycosylated residues. In red: residues of particular interest. EC, Extracellular; IV, Intravesicular; IC, Intracellular. (This figure was built using http://wlab.ethz.ch/protter/start/).

A photoaffinity labeling study revealed that SV2A, SV2B and probably SV2C are able to bind adenine nucleotides, particularly adenosine triphosphate (ATP) and nicotinamide adenine dinucleotide (NAD). SV2A has two binding sites located in the N-terminus: upstream the first TM domain and in the large intracytoplasmic loop (Figure 4). Although this observation may bring SV2A closer to the MFS family, neither ATP nor NAD transport activities were detected (Yao and Bajjalieh, 2008). Interestingly, SVOP is also able to bind adenine nucleotide (particularly NAD) via its C-terminal extremity (Yao and Bajjalieh, 2009) and Glut1 (a glucose transporter, another MFS member) features a nucleotide binding site as well. The site is located around TM domains 8 and 9 and negatively regulates the glucose transport activity of Glut1 as it allows Glut1 to be active only if ATP is depleted (Levine et al., 1998, 2002). Like Glut1, SV2 proteins could be regulated or, alternatively, could need adenine nucleotide bindings to perform their functions. As the majority of experimental studies of SV2A functions were performed as close to physiological conditions as possible, it is more likely that the adenine binding has a positive action on SV2A functions than the opposite. The presence of a nucleotide binding site on SV2A could provide a modulatory link between energy levels of the cell and its vesicle secretion mechanism.

Another feature of SV2A, and SV2 paralogs in general, is that they are involved in botulinum neurotoxins A and E (BoNT/A, BoNT/E) entrance into the cell. They are not the only synaptic protein involved in BoNT entrance, as SYT1 and SYT2 are also receptors for BoNT/B and BoNT/G neurotoxins (Verderio et al., 2006). BoNTs are a family of compounds produced by the bacteria *Clostridium botulinum* and are able to interfere with neurotransmission. Depending on the sub-type, the toxin will cleave the synaptosome-associated protein (SNAP-25), vesicle-associated membrane protein (VAMP or synaptobrevin) or syntaxin (Tighe and Schiavo, 2013). Cultured hippocampal neurons invalidated for *SV2A* and *SV2B* do not bind BoNT/A. Binding is restored by re-expression of SV2A, SV2B or SV2C and is mediated by the luminal domain (L4). SV2C exhibits the highest affinity for BoNT/A (Dong et al., 2006). More precisely, one glycosylated asparagine present in the center of the interface between SV2 and the toxin (residue 573 in SV2A, 516 in SV2B and 559 in SV2C) seems to be implicated in BoNT/A’s mode of entry (Mahrhold et al., 2016; Yao et al., 2016). Interaction studies of SV2C with BoNT/A suggest an efficient and rapid binding/unbinding of the pair mediated by the backbone of SV2C rather than a side-chain interaction, as Syt1 and Syt2 do for BoNT/B and G. Interestingly, the binding of SV2C to BoNT/A is facilitated at pH 5, close to SV conditions, whereas the binding of Syt2 with BoNT/B is pH-independent (Weisemann et al., 2016). BoNT/A, BoNT/E failing to penetrate *SV2A/B* DKO neurons appears to be quite logical as the interaction of BoNT/E is mediated by the L4 domain of SV2A and B exclusively. It is worth noting that chimeric SV2C-L4 domain does not bind BoNT/E and displays a higher molecular weight, suggesting additional glycosylation sites that might counteract BoNT/E interaction. Moreover, glycosylation at N573 of SV2A is essential for BoNT/E binding (Dong et al., 2008). Interestingly, a single-point mutation of SV2A N-glycosylation site produces a protein that is correctly localized to synapses and properly recycled, suggesting that the failed internalization of BoNT/E is due to an impaired binding (Kwon and Chapman, 2012). Finally, SV2 proteins are also capable of BoNT/D binding by an unknown mechanism independent of the L1, L3 or L4 domain (Peng et al., 2011).

## SV2A in The Pathophysiology of Neurological Diseases

### SV2A and Epilepsy

Because *SV2A* KO mice display spontaneous and violent seizures, and as the anti-epileptic drug Levetiracetam (LEV—also known as Keppra^©^) targets SV2A (Lynch et al., 2004), many studies have addressed the possible role(s) of SV2A in various pathological conditions, particularly in epilepsy pathophysiology.

Thanks to the availability of resected tissues from human brains after surgical treatments for pharmacologically-resistant epilepsies, several teams had the opportunity to analyze SV2A expression in such conditions. In temporal neocortices from patients with intractable temporal lobe epilepsy (ITLE), western blot quantifications revealed that SV2A is reduced by 40% compared to human brains lacking an epileptic focus (Feng et al., 2009). A comparable decrease (30%) is found in resected hippocampi from patients suffering from TLE combined with hippocampal sclerosis (HS). This is shown by a reduced labeling in the hippocampus except in the inner layer of the DG (van Vliet et al., 2009). In addition, resected tissues from two other causes of ITLE, focal cortical dysplasia (FCD) or tuberous sclerosis complex (TSC), also showed a reduction of SV2A expression. If FCD or TSC were combined with HS, SV2A was also reduced in the hippocampus (Toering et al., 2009). Interestingly, mesial temporal sclerosis (MTS) type 1A, also known as “classical hippocampal sclerosis”, presents both a downregulation of SV2A and an upregulation of SV2C (Crèvecœur et al., 2014). A similar finding has been made *in vitro* in chromaffin cells as compensation of SV2C in *SV2A* KO cells reaches almost wild-type levels of total SV2 proteins (Xu and Bajjalieh, 2001). This is of a particular interest in the context of TLE as the majority of epileptic foci occur near or inside the hippocampus where SV2C could potentially compensate for SV2A absence.

As a significant proportion of patients with glioma experiences focal epilepsy, the presence of SV2A was also investigated in tumoral and peritumoral tissues. Surprisingly, the level of expression was identical in patients suffering from epilepsy and in those who didn’t (de Groot et al., 2010). This challenges the role of SV2A in epileptogenesis, at least in these cases. It is worth noting that the first homozygous mutation of *SV2A* to be described in human was recently associated with intractable epilepsy, involuntary movements, microcephaly and developmental and growth retardations (Serajee and Huq, 2015). Both parents carry a variant allele, implying a recessive status, while no other mutations in the exome could explain the child’s phenotype.

All these observations highlight a potential neuroprotective role for SV2A, as its absence appears to facilitate the progression of epilepsy. In the hippocampus, a possible combination effect of unbalanced levels of SV2A and SV2C could also be implicated. However, since neuronal losses can be observed in TLE, further studies are needed to clarify the exact role of SV2A in epileptic pathophysiology.

From an experimental point of view, different animal models have also been studied. In chronic epileptic rats resulting from maximal electroshock (MES), SV2A levels decreased by 30%, which is similar to the reduction observed in human patients suffering from TLE-HS. In contrast, no difference could be seen between controls and rats which did not develop progressive forms of epilepsy after MES induction. More precisely, 1 day after status epilepticus (SE) occurred in rats, SV2A labeling faded out in the inner layer and the hilus of the DG. One week after SE, SV2A was reduced in CA regions and could barely be observed in DG. In chronic epileptic rats, SV2A levels remain reduced in almost all layers of the hippocampus even 6–8 months post-induction (van Vliet et al., 2009). In spontaneous epileptic rats (SER) which exhibit spontaneous and absence seizures without any external stimulation(s), SV2A levels are lower in almost all cortical regions and in the inner molecular layer of the hippocampus. Moreover, Syt1 levels roughly follow the same pattern. Interestingly, analysis of neuronal densities revealed no significant differences between SER and control rats (Hanaya et al., 2012), excluding a correlation between cell loss and SV2A decrease.

Contrasting with those observations, SV2A levels in the hippocampus of electrically kindled rats were significantly increased, as well as 7S SNARE complex expression (Matveeva et al., 2007). Those elevated levels lasted for at least 1 year (Matveeva et al., 2008). SV2A immunoreactivity was also increased in hippocampi of PTZ-kindled mice while no change was observed in the cortex or cerebellum. More precisely, higher SV2A levels were localized in the hilus of DG, together with GAD67 immunoreactivity, a GABAergic neuron marker (Ohno et al., 2009, 2012). The authors hypothesize that the increased concentration of SV2A in inhibitory neurons could counterbalance seizure occurrences.

It is important to note that, as different types of epilepsy exist, all animal models do not have the same characteristics. This might explain the striking differences found in SV2A levels. MES rat constitute a model close to SER as they keep experiencing seizures (11 seizures per day in average), several months after the last stimulation (van Vliet et al., 2009). The lower levels of SV2A in both models could corroborate the hypothesis that SV2A reduction is implicated in epilepsy progression and/or maintenance. In contrast, electrically-kindled rats and PTZ-kindled mice models where higher levels of SV2A are found, experience “evoked” seizures in response to convulsing chemicals or mild electrical stimulations rather than real spontaneous seizures (Sharma et al., 2007).

In 1999, Levetiracetam (LEV—commercial name: Keppra^©^) was approved by the Food and Drug administration as a novel anti-epileptic drug. It is particularly useful in patients suffering from partial seizures, patients less responsive to conventional drugs or showing risks of drug interactions (Hovinga, 2001). Even if the exact mechanism by which LEV acts on seizures is still undefined (we don’t even known if LEV is an agonist or antagonist of SV2A), we know that it binds to SV2A and that this binding is required for its anti-epileptic action (Lynch et al., 2004). Implicated residues in this binding are depicted in Figure 4 (Shi et al., 2011; Lee et al., 2015). It is worth noting that LEV is able to normalize under- and over-expression of SV2A in autaptic hippocampal neurons by controlling the concentration of SV2A in the synapse (Nowack et al., 2011). More recently, other compounds were added to the racetam family—Brivaracetam, Selectracetam—and show promises for epilepsy therapeutic control. Those molecules also bind SV2A (Gillard et al., 2011; Rogawski, 2016). As the link between SV2A and LEV was very recently extensively reviewed (Löscher et al., 2016), we will not further elaborate.

### SV2A and Other Neurological Diseases

Besides epilepsy, SV2A could also be involved in the pathophysiology of other neurological diseases. In 1996, SV2A was identified in dystrophic neurites around neuritic plaques of Alzheimer’s disease (AD) patients and normal aged brains. In contrast, no traces of SV2A were found in tangles, amyloid deposits or diffuse plaques in AD or normal aged brains (Snow et al., 1996). When it was demonstrated that hyper-excitability of hippocampal neurons could play an important role in the development of AD (Bookheimer et al., 2000; Dennis et al., 2010; Quiroz et al., 2010), the effect of LEV on amnestic mild cognitive impairment (aMCI) was investigated. A first study revealed that under low doses treatment, LEV was able to normalize hippocampal activation level and seemed to improve cognitive tasks (Bakker et al., 2012). In addition, immunoprecipitation studies of brain extracts showed an interaction between Amyloid Beta A4 Precursor Protein-binding Family B Member 1 (FE65) and SV2A (Nensa et al., 2014). Co-transfection of FE65 and SV2A modifies the FE65 distribution in the cytoplasm, suggesting an effect of SV2A on FE65 intracellular targeting. In parallel, combined proteomic and transcriptomic studies of brain organelles show that SV2A was 22 times more expressed in mitochondria compared to microsomes and the cytosol (Kislinger et al., 2006). Mitochondria dysfunction is another parameter that can take part in AD development (Swerdlow, 2012). Interestingly, SV2A was also localized in mitochondria of aging and late-onset AD cell models (Stockburger et al., 2016). In the same model, positive effects of LEV treatment on the mitochondrial fission and fusion balance were abolished if SV2A expression was invalidated by siRNA.

SV2B also seems to be affected in AD. *SV2B* gene expression is down regulated in hippocampi and neocortices of AD patients compared to control (Gómez Ravetti et al., 2010; Tan et al., 2010). Moreover, *SV2B* (along with other genes involved in synaptic plasticity, vesicle fusion or docking) is down-regulated in hippocampi of AD cases with expression of APOE alleles associated with higher risks of early AD onset compared to other AD patients (Xu et al., 2006). On the contrary, neurons exposed to amyloid beta peptides (Aβ) specifically overexpress an mRNA transcript variant of *SV2B* with an elongated 3′UTR. The authors suggest that this change could be involved in neurodegeneration progression (Heese et al., 2001). This observation is confirmed by the fact that in *SV2B* KO mice, the effect of an oligomeric preparation of Aβ_25–35_ is strongly reduced (Detrait et al., 2014).

Schizophrenia (SCZ) is another disorder that has been recently suspected to involve SV2A in its pathophysiology pathway. Genomic analysis of several cerebellar cortices from patients suffering from SCZ revealed an alteration of numerous synaptic transport proteins, SV2A being one of them (Mudge et al., 2008). Furthermore, a single nucleotide polymorphism (SNP) in the *SV2A* gene region was found to be significantly associated with SCZ. *SV2A* gene is surrounded by histone gene clusters, known to be linked to SCZ. Therefore, further studies are needed to clarify which gene is influenced by the SNP (Mattheisen et al., 2012). Interestingly, a novel drug devoid of anticonvulsant properties (UCB0255) and acting as a negative modulator of SV2A, seems to increase the cognitive capacity in the sub-chronic phencyclidine (PCP) rat model. This rat line exhibits typical SCZ parameters like low prefrontal dopamine, low prefrontal metabolism and enduring cognitive deficits (Laruelle et al., 2014).

## Could All SV2 Proteins Endorse The Same Role?

The discovery circumstances of SV2 proteins give the illusion that SV2 proteins are isoforms. It is now known that SV2 proteins have their attributed genes on different chromosomes (Table 1) and that they arose from duplication events that may have taken place around the apparition of vertebrates, making them paralogs (Figure 4).

At first, the absence of any phenotype in *SV2B* KO mice suggested that the role of SV2B was different from the one of SV2A. But the fact that SV2B is able to rescue synaptic depression in cultured neurons proves that SV2A and SV2B share at least one role in neurotransmission (Nowack et al., 2010). This is surprising because *SV2B* KO mice do not exhibit even mild neurotransmission problems. Indeed, they are usually used as controls in *SV2A/B* DKO experiments. Several SV2B features could explain this situation. First, the distribution pattern of SV2B is slightly narrower compared to SV2A. If the severity of *SV2A* KO phenotype is driven by the fact that it impacts virtually all synapses of the brain, it may seem logical that *SV2B* KO mice would be less or not affected. On the other hand, seizures could typically arise after the development of an epileptic focus corresponding to either localized or broader brain regions: no proof has been provided yet to demonstrate whether or not SV2A could induce partial or simple seizures. It has been proposed that the normal phenotype of *SV2B* KO mice is due to an overall lower expression level of SV2B compared to SV2A (Nowack et al., 2010). Indeed, in the vast majority of the brain, SV2A reaches a higher expression level than SV2B, but more importantly SV2B is expressed primarily in glutamatergic synapses whereas SV2A can be found in both networks. This means that SV2A and SV2B could compensate each other’s absence only in glutamatergic synapses. Rod photoreceptor cells are one of the few neuronal cell types where SV2A has a faint expression whereas SV2B is highly concentrated. *SV2B* KO mice display reduced b-wave amplitude at rod synapses, indicating that SV2B loss could induce a decrease in neurotransmission (Wang et al., 2003; Morgans et al., 2009) as SV2A absence does. Finally, cortical and hippocampal glutamatergic networks seem to be less affected by SV2A absence than GABAergic ones and the combined absence of SV2A and B does not seem to drastically alter neurotransmission. For this reason, a SV2 defect affecting the GABAergic networks appears more effective than the same defect targeting the glutamatergic networks.

Given the peculiar distribution pattern of SV2C, this paralog was immediately considered to differ from the two others. Very few studies have been performed on SV2C but the generation of *SV2C* KO mice will provide a useful tool to assess its potential role (Dardou et al., 2011). These mice present an upregulation of tyrosine hydroxylase mRNA combined with downregulation of encephalin mRNA. After 6-hydroxydopamine and MPTP-induced lesions (two experimental models of Parkinson’s diseases), *SV2C* mRNA levels increased significantly. *SV2C* KO mice seem to present slightly less exploratory and slightly more anxious behavior (Dardou et al., 2013). In parallel, an association study on the genome of human and *Drosophila* revealed that SV2C could be implicated in PD through its potential role in nicotine neuroprotection (Hill-Burns et al., 2013). More recently, a team showed that SV2C interacts with α-synuclein in mouse homogenates and that *SV2C* KO mice exhibits reduced level of dopamine release. Moreover, brain sections of patients suffering from Parkinson’s diseases also seem to display more SV2C-positive labels than normal aged brains (Dunn et al., 2017).

## Conclusions

Even if more and more data are gathered on SV2A, its exact function is still a matter of debate.

In hippocampal or cortical neuronal cell populations, loss of SV2A leads to synaptic facilitation and to the reduction of post-synaptic currents amplitude at least in excitatory synapses. The possibility that the RRP of SVs is affected in *SV2A* KO is still discussed (Custer et al., 2006; Chang and Sudhof, 2009). If indeed this is the case, one could recall a priming defect, reinforced by the reduction of SDS resistant SNARE complex as seen in SV2A-deficient chromaffin cells or by the combined increase of 7S SNARE complex and SV2A as seen in PTZ-induced mice (Xu and Bajjalieh, 2001; Matveeva et al., 2008). If it is not the case, a decreased capacity to trigger synaptic release could be evoked, which is consistent with the Syt1 recycling function of SV2A in parallel with Stn2 (Nowack et al., 2010; Yao et al., 2010; Kaempf et al., 2015). Moreover, the fact that the absence of SV2A induces an increased recycling of Syt1 different from the clathrin-mediated recycling after a train of stimulations (Zhang et al., 2015), could participate to the facilitation phenotype. This increased recycling is also produced by the absence of Stn2, as well as following mutation of the binding site on SV2A, Syt1 or the overexpression of Syt1 (Kononenko et al., 2013; Zhang et al., 2015). The distribution of Stn2 has never been deeply investigated but the protein seems to be expressed in most parts of the brain and particularly in the hippocampus (Walther et al., 2001, 2004), matching the ubiquitous distribution of SV2A.

The fact that SV2A-R231Q rescues neurotransmission defects in SV2A KO without correcting the accumulation of Syt1 usually observed in SV2A absence, suggests that SV2A main function is not the recycling of Syt1 (Nowack et al., 2010). Finally, glycans present on the L4 domain of SV2A could serve as vesicular matrices but strong evidence supporting this affirmation is lacking (Reigada et al., 2003).

In mice, absence of SV2A leads to early death after epileptic seizures and seems to cause a reduction of inhibitory post-synaptic current frequencies and an augmentation of excitatory post-synaptic current frequencies, reversing the normal balance between those two inputs (Venkatesan et al., 2012). On the other hand, rats with a mutated form of SV2A are more susceptible to PTZ treatment and present a reduction of depolarization-induced GABA release in the hippocampus and the amygdala but no changes in depolarization-induced glutamate release (Tokudome et al., 2016a,b), linking again specifically SV2A with the GABAergic network. This is truly surprising as the presence of SV2A in glutamatergic synapses has been proven by a broad range of techniques. Still in any case, the fact that no change in glutamate release was observed is surprising as excitatory frequencies seem to be increased due to inhibitory frequency reductions.

In some epileptic patients and “long-term” animal models of epilepsy, a significant reduction of SV2A is observed, supporting the idea that SV2A could endorse a role in epilepsy progression (Feng et al., 2009; Toering et al., 2009; van Vliet et al., 2009; Hanaya et al., 2012). In “short-term” animal models of this disease, SV2A is overexpressed suggesting a compensatory phenomenon (Matveeva et al., 2007, 2008; Ohno et al., 2009, 2012).

More and more observations bring SV2A and SV2B closer as they appear to compensate each other *in vitro* and as both seem to be implicated in AD. Based on observations of *SV2A*, *SV2B* and *SV2A/B* KO mice, it seems that in glutamatergic neurons the presence of SV2B allows to compensate SV2A absence. The low level of SV2B in GABAergic cells would then favor a link between SV2A loss and seizures development. As an alternative explanation, one (or several) unknown protein(s) could be able to compensate SV2A and SV2B absences in glutamatergic synapses only, reducing the impact of SV2 absence on this network compared to the GABAergic system. This wouldn’t be the case in retinas, explaining the severe defect associated with SV2B absence in this structure. It is unlikely that this role could be assumed by *Stn2* because a *SV2A* mutants do elicit normal Syt1 recycling and still induce neurotransmission problems (Nowack et al., 2010).

There are a lot of answers needed to deepen the understanding of SV2 functions and mechanisms. First, given the number of common points between Stn2 and SV2A, it would be interesting to compare their expressions, searching for a correlation in Stn2/SV2A ratios between different cell types, synapse types or brain regions. Second, considering the fact that SV2A overexpression in cultured neurons presents the same neurotransmission modifications and/or impairments than its absence (Nowack et al., 2011), overexpressing SV2A in animal models could be interesting and provide information on a largely underexplored field. Next, comparing the proteomic content of vesicles obtained from glutamatergic synapses in different parts of the brain could shed light on the potential compensation of SV2A and/or SV2B absence in glutamatergic neurons. Finally, following Tukodome’s group recent articles, the impact of SV2A absence in different cell types on seizures onset is still unclear and requires additional observations. To this aim, a new mouse model based on the cre-lox system (Menten-Dedoyart et al., 2016) will allow the invalidation of SV2A in specific inhibitory neuron populations and a comparison of obtained phenotypes with those where SV2A is absent in excitatory neuron populations. This research effort could also bring answers and explanations to several electrophysiological differences observed in SV2A KO phenotypes.

## Author Contributions

OB: manuscript writing; PVA, JSG, OBB, PL, RF and BR: manuscript revision.

## Funding

This work was supported by Special Funds and ARC of the University of Liège, Grants from the FSR-FNRS and the Fond Léon Fredericq.

## Conflict of Interest Statement

The authors declare that the research was conducted in the absence of any commercial or financial relationships that could be construed as a potential conflict of interest.

## Footnotes

^1^https://blast.ncbi.nlm.nih.gov/Blast.cgi
